# Predictors and outcome of patients with acute respiratory distress syndrome caused by miliary tuberculosis: a retrospective study in Chongqing, China

**DOI:** 10.1186/1471-2334-12-121

**Published:** 2012-05-20

**Authors:** Wang Deng, Min Yu, Hilary Ma, Liang An Hu, Gang Chen, Yong Wang, Jia Deng, ChangYi Li, Jin Tong, Dao Xin Wang

**Affiliations:** 1 Department of Respiratory Medicine, Second Affiliated Hospital of Chongqing Medical University, Chongqing, China; 2 Department of Medicine, New York University Langone Medical Center, New York, USA; 3 Department of Respiratory Medicine, First Affiliated Hospital of Chongqing Medical University, Chongqing, China; 4 Department of Respiratory Medicine, Chongqing Chest Hospital, Chongqing, China; 5 Department of Respiratory Medicine, Chongqing Pulmonary Hospital, Chongqing, China

**Keywords:** Acute respiratory distress syndrome, Miliary tuberculosis, Predictors, China

## Abstract

**Background:**

Miliary tuberculosis (TB) is an uncommon cause of acute respiratory distress syndrome (ARDS) with a high mortality. The aim of the present study was to evaluate the clinical characteristics, predictors and outcome of patients with ARDS caused by miliary TB.

**Methods:**

A retrospective study was conducted among patients with a diagnosis of ARDS with miliary TB in four hospitals from 2006 to 2010. Medical records and laboratory examinations of these patients were taken during the first 24 h of admission.

**Results:**

Eighty-five patients with miliary TB developed ARDS, 45 of whom survived (52.9%). The median age was 36.6 ± 12.5 years with 38 males (44.7%). Diabetes mellitus (DM) was the most common underlying disease (18.8%).ICU mortality was 47.1%. The time from admission to anti-tuberculosis therapy was 4.5 ± 2.0 days. Mean duration of mechanical ventilation was 8.5 ± 3.0 days in all patients. Duration of time to diagnosis, time from diagnosis to mechanical ventilation, and time to anti-tuberculosis therapy were significantly shorter in survivors than those in non-survivors. Diabetes mellitus (OR 5.431, 95%CI 1.471-20.049; P = 0.005), ALT (70-100U/L, OR 10.029, 95%CI 2.764-36.389; P = 0.001), AST (>94U/L,OR 8.034, 95%CI 2.200-29.341; P = 0.002), D-dimer (>1.6mg/L, OR 3.167, 95%CI 0.896-11.187; P = 0.042), hemoglobin (<90g/L, OR 14.824, 95%CI 3.713-59.179; P = 0.001), albumin (<25g/L, OR 15.896, 95%CI 3.975-63.566; P = 0.001) were independent predictors of ARDS development in the setting of miliary TB.

**Conclusions:**

Accurate diagnosis, early initiation of anti-tuberculosis therapy and mechanical ventilation are important for the outcome of patients with ARDS caused by miliary TB. DM, ALT, AST, D-dimer, hemoglobin, and albumin are independent predictors of ARDS development in patients with miliary TB.

## Background

Tuberculosis(TB) remains a major and global health disease[1,2]. Recent studies have shown the link between acute respiratory distress syndrome (ARDS) and pulmonary TB[3,4]. Pulmonary TB complicated by ARDS is often found in the setting of miliary TB[3-6]. Most reports on ARDS caused by miliary TB are small numbers of patients in the case reports. Compared with miliary TB alone, miliary TB with ARDS portends a higher mortality of 33-90% [7-9]. Duration of miliary TB beyond 20 days tends to markedly increase the risk of ARDS [10]. It is very important for recognition of ARDS caused by miliary TB.

Despite being a well-documented entity, miliary TB complicated by ARDS remains a challenging diagnosis due to its variable clinical manifestations and low morbidity. Some predictors such as AST, and ALT[4,10] in miliary TB with ARDS have drawn our attention. Numerous case reports have mentioned that TB with ARDS is more common than TB complicated by anemia[4,11] and hypoproteinemia[6,11]. Thus, identifying the predictors of miliary TB associated with ARDS can play an important role in diagnosis and therapy. The aim of the present study was to determine the predictors and their impact on outcome based on a retrospective analysis of patients with ARDS caused by miliary TB in four hospitals of Chongqing in China, with the hope that this will contribute to a better understanding and improved management of the disease.

## Methods

### Study design

Over a 5-year period (2006.03.31-2010.03.31), 16238 patients were admitted with a diagnosis of pulmonary TB in the respiratory departments of Second Affiliated Hospital of Chongqing Medical University, First Affiliated Hospital of Chongqing Medical University, Chest Hospital and Pulmonary Hospital in Chongqing, China. Of these, 471 patients were diagnosed with miliary TB. Five patients were excluded for further analysis due to transfer to another hospital within 2 days. Of the remaining 466 patients, 85 patients developed ARDS were admitted to the ICU during the study period. The patients with ARDS caused by miliary TB were divided into survivor group(n = 45) and non-survivor group(n = 40).

Our research protocol was approved by the institutional review boards of all participating institutions.

### Data collection

The original case records including clinical profiles and laboratory parameters at admission were gathered from the registration departments. Age, sex, past medical history, underlying diseases, PaO2/FiO2, time from diagnosis to mechanical ventilation, time from admission to anti-tuberculosis therapy, duration of time to diagnosis, and lengths of stay in the ICU and in the hospital were collected. Acute Physiology and Chronic Health Evaluation (APACHE) III score were calculated on the day of diagnosis with ARDS. The results of acid-fast bacilli(AFB) smears, culture of respiratory specimens including sputum, tracheal aspirate or bronchoalveolarlavage (BAL)fluid and histopathological examination were recorded. Laboratory data including aspartate aminotransferase (AST), alanine aminotransferase (ALT), erythrocyte sedimentation rate (ESR), D-dimer, hemoglobin, and albumin were taken during the first 24 h of admission.

### Definition

The diagnosis of miliary TB was made based on: equality of the size, distribution, density miliary-like nodules bilaterally diffused on chest radiography by at least 2 independent of radiologists. Pulmonary TB was confirmed by at least one of the three following criteria:1)positive AFB smear and/or culture for M. tuberculosis from respiratory specimes;2) histopathological identification of TB granulomain in biopsied tissues of lung, and/or pleura;3)clinical and radiographic improvement after anti-tuberculosis treatment[4]. The diagnosis of ARDS was made in accordance to the diagnostic criteria of European-American Consensus Conference on ARDS[12]:acute in onset with PaO_2_/FiO_2_ ≤ 200mmHg, bilateral infiltrates seen on chest radiograph, and pulmonary artery wedge pressure ≤ 18 mm Hg. Survivors were defined as patients who survived to discharge from hospital. Patients with human immunodeficiency virus (HIV), H1N1 Influenza A,and procalcitonin (PCT) positive were excluded.

### Statistical analysis

Continuous variables were presented as mean ± standard deviation(SD) or median and compared using an unpaired *t*-test and the Mann–Whitney *U* test. Categorical variables were compared using the Chi-squared test. Multivariate logistic regression analysis was performed to determine the predictors. All statistical analysis were performed using SPSS 13.0. *P* <0.05 was considered significant.

## Results

### Patient characteristics

Patients’ clinical characteristics and outcome are presented in Table 1. All patients experience typical symptoms of miliary TB. Diabetes mellitus (DM) was the most common underlying disease (18.8%). Thirty-three (38.8%) patients had been initially misdiagnosed with viral pneumonia, hypersensitivity pneumonitis, acute interstitial pneumonia, fungal pneumonia, alveolar cell carcinoma, or meningitis for a median of 7.2 ± 3.4 days from admission. The diagnosis of miliary TB was established by AFB smear and/or culture of respiratory specimens (including sputum,tracheal aspirate or BAL fluid) in 61 patients(71.8%), by histopathological examination of tissue biopsy in 11 patients(12.9%), and by clinicoradiological diagnosis in 13 patients(15.3%). Bacterial isolate drug sensitivity data were available from 43 patients (50.6%), 3(3.5%) demonstrated at least single drug resistance.

**Table 1:** **Clinical characteristics, investigations and outcome of patients with ARDS caused by miliary TB (n = 85**)

Parameters	Results
Age (years)	36.6 ± 12.5
Sex (male)	38(44.7%)
Clinical findings	
Fever, cough, dyspnoea, weight loss	85 (100)
Diabetes mellitus	16 (18.8)
Past history of tuberculosis	9(10.6)
Hepatitis B	8 (9.4)
Pregnancy	3 (3.5)
Heart disease	3(3.5)
Cerebrovascular disease	2(2.3)
Investigations	
APACHE III score	71.6 ± 21.9
PaO2/FiO2(mmHg)	146.4 ± 34.5
Outcome	
Duration of symptoms (days)	16.1 ± 5.2
Misdiagnosis for other diseases	33 (38.8)
Time to diagnosis (days)	7.2 ± 3.4
Length of stay in the ICU(days)	15.3 ± 3.5
Length of stay in the hospital(days)	26.7 ± 4.6
Duration of mechanical ventilation (days)	8.5 ± 3.0
Time from admission to anti-tuberculosis therapy (days)	4.5 ± 2.0
Glucocorticoids therapy	35 (41.2)
ICU mortality	40(47.1)

Data are presented as mean + SD or n(%).

### Hospital course and outcome

The time from admission to anti-tuberculosis therapy was 4.5 ± 2.0 days. All 85 patients with ARDS were prescribed anti-tuberculosis medication consisting of isoniazid, rifampicin, ethambutol, and pyrazinamide. Mechanical ventilation was necessary in all 85 patients. Thirty-eight patients(44.7%) required invasive mechanical ventilation while the rest were given non-invasive mechanical ventilation with BiPAP. Mean duration of mechanical ventilation was 8.5 ± 3.0 days with ICU mortality of 47.1%. Thirty-five patients (41.2%) received glucocorticoid therapy (methylprednisolone:80 mg/day) intravenously for a maximum of 5 days when anti-tuberculosis therapy was started. The use of glucocorticoids was associated with a mortality of 22.9% (8/35) compared with 76.0% (38/50) in those who were not treated with glucocorticoids. Comparison between patients with miliary TB developing ARDS and patients with miliary TB alone are shown in Table 2. Comparison between survivors and non-survivors of ARDS patients are shown in Table 3. Duration of time to diagnosis, time from diagnosis to mechanical ventilation, and time from admission to anti-tuberculosis therapy were significantly shorter in survivors than non-survivors. Also, DM, ALT, AST, D-dimer, hemoglobin, and albumin showed significant difference between the survivor group and non-survivor group. The 3 pregnant patients underwent termination of pregnancy, one of whom died of respiratory failure.

**Table 2:** Comparisons of demographic parametres, clinical and laboratory characteristics between ARDS group and non-ARDS group

	ARDS(n = 85)	non-ARDS(n = 381)	P-value
Age(years)	36.6 ± 12.5	38.1 ± 11.9	0.892
Sex(male)	38(44.7%)	113(40.2%)	0.710
ALT (U/L)	73.7 ± 20.9	30.9 ± 25.8	0.001
AST (U/L)	94.7 ± 18.2	35.7 ± 23.1	0.001
ESR(mm/the first hour)	40.3 ± 18.8	42.8 ± 28.6	0.653
D-dimer (g/L)	1.2 ± 0.5	0.3 ± 0.2	0.001
Hemoglobin (g/L)	83.5 ± 16.2	125.4 ± 35.4	0.011
Albumin (g/L)	29.3 ± 3.7	40.2 ± 14.1	0.001

Data are presented as mean + SD or n (%).AST: aspartate aminotransferase; ALT: alanine aminotransferase. ESR:erythrocyte sedimentation rate.

**Table 3:** Comparisons of demographic parametres, clinical and laboratory characteristics between survivors and non-survivors of ARDS patients

	Survivors(n = 45)	Non-survivors(n = 40)	*P*-value
Age(years)	35.8 ± 9.5	37.1 ± 14.2	0.899
Sex(male)	20(44.4)	18(45)	0.745
DM	4(8.89)	12(30)	0.017
APACHE III score	70.4 ± 23.6	72.8 ± 19.1	0.856
PaO2/FiO2(mmHg)	149.5 ± 37.8	143.7 ± 31.9	0.648
Time to diagnosis (days)	3.2 ± 2.7	11.8 ± 4.1	0.002
Time from diagnosis to mechanical ventilation (days)	2.5 ± 3.4	8.6 ± 5.3	0.034
Time from admission to anti-tuberculosis therapy (days)	2.8 ± 1.6	6.9 ± 3.8	0.046
Length of stay in the ICU(days)	19.3 ± 4.9	12.4 ± 3.2	0.267
Length of stay in the hospital(days)	29.4 ± 5.6	17.3 ± 4.2	0.366
ALT (U/L)	56.2 ± 19.6	93.2 ± 22.4	0.038
AST (U/L)	63.8 ± 18.1	118.2 ± 21.4	0.019
ESR(mm/the first hour)	40.0 ± 18.1	40.6 ± 18.9	0.714
D-dimer (g/L)	0.6 ± 0.8	1.8 ± 0.6	0.017
Hemoglobin (g/L)	92.7 ± 14.8	76.2 ± 20.9	0.032
Albumin (g/L)	34.6 ± 3.9	23.9 ± 3.2	0.027

Data are presented as mean ± SD or n(%).AST: aspartate aminotransferase; ALT: alanine aminotransferase; ESR:erythrocyte sedimentation rate; DM:diabetes mellitus.

### Predictors of ARDS development caused by military TB

Positive likelihood ratio were performed to analyze sensitivity and specificity of the predictors level(the greater the ratio the greater probability of true positive in a positive result) (Table 4). On multivariate logistic regression analysis, presence of DM(OR 5.431, 95%CI 1.471-20.049; P = 0.005),ALT (70-100U/L, OR 10.029, 95%CI 2.764-36.389; P = 0.001), AST (>94U/L,OR 8.034, 95%CI 2.200-29.341; P = 0.002), D-dimer (>1.6mg/L, OR 3.167, 95%CI 0.896-11.187; P = 0.042), hemoglobin (<90g/L, OR 14.824, 95%CI 3.713-59.179; P = 0.001), albumin (<25g/L, OR 15.896, 95%CI 3.975-63.566; P = 0.001) were independent predictors of ARDS development caused by military TB.

**Table 4:** The levels of predicators for ARDS development caused by military TB

Predicators	Sensitivity	Specificity	Positive likelihood ratio
ALT			
Level1	41.9%	4.8%	0.44
Level 2	19.4%	96.3%	5.24
Level 3	25.8%	99.7%	86.00
Level 4	12.9%	99.1%	14.33
AST			
Level1	32.2%	8.5%	0.35
Level 2	22.6%	96.0%	5.65
Level 3	29.0%	96.0%	7.25
Level 4	16.1%	99.4%	26.83
D-dimer			
Level1	64.5%	23.7%	0.84
Level 2	25.8%	76.8%	1.11
Level 3	3.2%	99.4%	5.33
Level 4	6.5%	100%	+∞
Hemoglobin			
Level1	22.6%	8.2%	0.25
Level 2	19.4%	93.5%	2.98
Level 3	22.6%	98.9%	20.55
Level 4	35.5%	99.4%	59.16
Albumin			
Level1	16.1%	5.6%	0.17
Level 2	58.1%	96.3%	15.70
Level 3	12.9%	98.6%	9.21
Level 4	12.9%	99.4%	21.50

ALT:Level 1:0-40U/L;Level 2:40-70U/L;Level 3:70-100U/L;Level 4: > 100U/L.

AST:Level 1: < 34U/L;Level 2:34-64U/L;Level 3:64-94U/L;Level 4: > 94U/L.

D-dimer:Level 1: < 0.34mg/L;Level 2:0.34-1.0mg/L;Level 3:1.0-1.6mg/L;Level 4: > 1.6mg/L;

Hemoglobin:Level 1:110-160g/L;Level 2:100-110 g/L;Level 3:90-100 g/L;Level 4: < 90 g/L;

Albumin:Level 1:35-50 g/L;Level 2:30-35g/L;Level 3:25-30 g/L;Level 4: < 25 g/L.

## Discussion

In the present study,our results demonstrated accurate diagnosis and early therapeutic management were crucial to optimizing outcome and DM, ALT, AST, D-dimer, hemoglobin, and albumin are independent predictors of ARDS development in patients with miliary TB. Tuberculosis remains a major cause of morbidity and mortality around the world, especially in developing countries [13,14]. According to the 13th annual tuberculosis report of the World Health Organization (WHO), there were an estimated 9.27 million new cases worldwide in 2007, an increase from 9.24 million in 2006[1]. Further, miliary TB is an uncommon cause of ARDS with a high mortality [4,15]. The current study was performed in Chongqing, the fourth central municipality of China, which has a prevalence rate of 0.54%, higher than the national average. In our study, the average age of patients with ARDS caused by miliary TB was younger than that previously reported due to the possible reason of host factors including region, race, and environment [3,4].

Mechanical ventilation is an important treatment for miliary TB complicated by ARDS. Prompt mechanical ventilation from the day of diagnosis can effectively improve outcome, which benefits for the management of ARDS caused by miliary TB. Also, accurate diagnosis and early anti-tuberculosis therapy are crucial to the treatment of miliary TB with ARDS. However, the high overall mortality is attributed to case mix, misdiagnosis, and severity of illness, which ultimately lead to the delay in the initiation of anti-tuberculosis therapy or mechanical ventilation.

Albumin plays an important role in regulating plasma osmolality. Hypoproteinemia accelerates fluid exudation, promotes alveolar edema, and contributes to ventilation-perfusion imbalance. Also, in infected mycobacterium tuberculosis, inflammatory cells accumulate in the alveolar spaces, releasing granular enzymes and oxidants which participate in local inflammation and overlapping reactions and damaging the alveolar basement which allows increase in cellular permeability that aggravates oxygen dysfunction and consequently causes ARDS [16,17]. If the process continues, cytokines activate an inflammatory cascade reaction and lead to other organs dysfunction, resulting in increases in AST and ALT. The changes in AST, ALT, and serum albumin were significantly different between survivor and non-survivor groups. The results showed that AST, ALT, and serum albumin could be independent predictors of ARDS development in miliary TB.

A number of studies have documented anemia associated with TB[18-20]. It is well established that erythropoiesis is suppressed by inflammatory mediators in anemia of chronic infection or inflammation and anemia caused by chronic infections including TB results from the effects of cytokines that mediate the inflammatory response,which may provides the development for ARDS[21-23]. As we know,the severity of anemia is mainly determined by hemoglobin level. The decrease in hemoglobin is assumed as a reflection of inflammation,which can explain the relationship between ARDS and hemoglobin in our study. This relationship is supported by our finding of hemoglobin being an independent predictor of ARDS development in miliary TB.

D-dimer is a fibrin degradation product formed during fibrinolysis, the process of breaking down a blood/fibrin clot. Our study showed that D-dimer was an independent predictor of ARDS development and mortality. Hyperglycemia is known to have a proinflammatory effect, and may be correlated with poor outcome in hospitalized/critically ill patients. Consistent with this correlation, odds ratio analysis after ARDS development identified DM as a risk factor.

To further understand the accurate levels of the predictors for predicting ARDS, we used stratified analysis for each index. Positive likelihood ratio was included in the study due to a combination of sensitivity and specificity to reflect the reality of the indicators. ALT(>70-100U/L), AST (> 94U/L), D-dimer (>1.6mg/L), hemoglobin (<90g/L), and albumin (<25g/L) at the time of admission were independent predictors for ARDS development in the setting of miliary TB. Simple miliary TB can damage organs, inducing mild increases in ALT and AST, although these incremental changes are too small to have any accuracy in predicting the occurrence of ARDS. However, when one develops ARDS, inflammatory mediators will seriously damage the organs, resulting in significantly higher increases in the indexes than those in simple miliary TB, which may translate into higher accuracy for predicting ARDS. Though ESR was significantly elevated in miliary TB, it did not reach statistical significance in association with ARDS. Our findings suggested that ESR has minimal value in predicting ARDS.

In addition, for patients with miliary TB who developed ARDS during pregnancy, there are few case reports with favorable outcome. Our data suggested that pregnancy was a risk factor for ARDS, but this clinical observation was limited by the small sample size. The efficacy of systemic corticosteroids is well-documented for several extrapulmonary complications of tuberculosis such as tuberculous meningitis and tuberculous pericarditis[24-28], as well as ARDS[29-32]. The role of glucocorticoids remains controversial in the management of miliary TB complicated by ARDS[33,34]. In our study, patients treated with glucocorticoids had a lower mortality than those who did not, which might suggest that methylprednisolone at a dose of 80 mg/day was given intravenously at the time when anti-tuberculosis therapy was started might be benefit for ARDS associated with miliary TB. The limitations of this study are studies with large numbers of patients may be required to validate the observations due to a relatively small sample size in the present study.

## Conclusions

In conclusion, the mortality of ARDS caused by miliary TB remains high. Accurate diagnosis and early therapeutic management of therapy including anti-tuberculosis agents and mechanical ventilation are crucial to optimizing outcome. DM, ALT, AST, D-dimer, hemoglobin, and albumin are independent predictors of ARDS development in patients with miliary TB.

## Competing interests

The authors declare that they have no competing interests.

## Authors’ contributions

WD,HM and DXW participated in the conception and design of the study. WD, MY,LAH,GC,YW,JD,CYL and JT performed the acquisition of data and the statistical analysis. WD drafted the manuscript. MY helped to draft the manuscript and coordination. HM and DXW participated in the revision of the manuscript. All authors have read and approved the final manuscript.

## Pre-publication history

The pre-publication history for this paper can be accessed here:

http://www.biomedcentral.com/1471-2334/12/121/prepub
